# Understanding barriers and facilitators to palliative and end-of-life care research: a mixed method study of generalist and specialist health, social care, and research professionals

**DOI:** 10.1186/s12904-024-01488-2

**Published:** 2024-06-25

**Authors:** Catherine Walshe, Lesley Dunleavy, Nancy Preston, Sheila Payne, John Ellershaw, Vanessa Taylor, Stephen Mason, Amara Callistus Nwosu, Amy Gadoud, Ruth Board, Brooke Swash, Seamus Coyle, Andrew Dickman, Andrea Partridge, Jaime Halvorsen, Nick Hulbert-Williams

**Affiliations:** 1 International Observatory On End-of-Life Care, Division of Health Research, Faculty of Health and Medicine, Lancaster University, Lancaster, UK; 2 Liverpool University, Liverpool, UK; 3 University of Huddersfield, Huddersfield, UK; 4 Lancaster Medical School, Lancaster University, Lancaster, UK; 5 Lancashire Teaching Hospitals NHS Foundation Trust, Preston, UK; 6 Chester University, Chester, UK; 7 The Clatterbridge Cancer Centre NHS Foundation Trust, Liverpool, UK; 8 Liverpool University Hospitals NHS Foundation Trust, Liverpool, UK; 9 NIHR Clinical Research Network North West Coast, Liverpool, UK; 10 Edge Hill University, Ormskirk, UK

**Keywords:** Palliative care, Palliative Medicine, Hospices, Surveys and Questionnaires, Research, Research Priorities, Health Services Research, Methodological Studies

## Abstract

**Background:**

Palliative care provision should be driven by high quality research evidence. However, there are barriers to conducting research. Most research attention focuses on potential patient barriers; staff and organisational issues that affect research involvement are underexplored. The aim of this research is to understand professional and organisational facilitators and barriers to conducting palliative care research.

**Methods:**

A mixed methods study, using an open cross-sectional online survey, followed by working groups using nominal group techniques. Participants were professionals interested in palliative care research, working as generalist/specialist palliative care providers, or palliative care research staff across areas of North West England. Recruitment was via local health organisations, personal networks, and social media in 2022. Data were examined using descriptive statistics and content analysis.

**Results:**

Participants (survey *n* = 293, working groups *n* = 20) were mainly from clinical settings (71%) with 45% nurses and 45% working more than 10 years in palliative care. 75% were not active in research but 73% indicated a desire to increase research involvement. Key barriers included lack of organisational research culture and capacity (including prioritisation and available time); research knowledge (including skills/expertise and funding opportunities); research infrastructure (including collaborative opportunities across multiple organisations and governance challenges); and patient and public perceptions of research (including vulnerabilities and burdens). Key facilitators included dedicated research staff, and active research groups, collaborations, and networking opportunities.

**Conclusions:**

Professionals working in palliative care are keen to be research active, but lack time, skills, and support to build research capabilities and collaborations. A shift in organisational culture is needed to enhance palliative care research capacity and collaborative opportunities across clinical and research settings.

**Supplementary Information:**

The online version contains supplementary material available at 10.1186/s12904-024-01488-2.

## Background

Palliative care provision should be informed by high quality research, so that clinical practice is underpinned by a robust evidence base. Improving the evidence base in palliative care is a ‘moral imperative’, with arguments highlighting that it is ethically important to offer effective treatments supported by an evidence base, and equally that futile treatments are avoided [1]. A principal focus of much of the research conducted to understand why developing the evidence base is difficult has focused on the specific challenges of recruiting patient and carer participants to palliative care research studies. Gatekeeping can be an issue, with staff concerned about overburdening vulnerable patients and carers, and feeling ill prepared to discuss research with potential participants [2–4]. This is despite evidence suggesting patients and families are willing to engage in research at the end of life [5–7]. Despite this readiness, there can be many reasons why patients and carers may not feel able to engage in research such as illness severity, symptom burden, misconceptions about palliative care, lack of cure and perceived therapeutic benefit, and study burden [8–10]. This can mean that many studies experience recruitment difficulties [11, 12]. Facilitators that may address some of these complex structural, cultural and personal barriers include dedicated research staff on site [3, 13], training on how to recruit to palliative care studies [14, 15], and improving communication with patients and their families to promote research participation, and within staff teams to address gatekeeping.

Researchers outside palliative care have chosen to explore the professional and organisational facilitators and barriers to conducting research [16, 17]. Less is known about the personal, professional, organisational, and structural barriers and facilitators to conducting palliative care research. Palliative care requires a multi-professional approach, and patients are cared for in a variety of settings, including hospitals, hospices, nursing homes and primary care. Palliative care research is historically under-funded in comparison to research that focuses on the prevention or cure of cancer and other life-limiting illnesses [18, 19]. There may also be challenges with access to staff with the relevant research expertise, and complicated or undeveloped governance arrangements particularly in settings outside statutory provision [20–23]. Research may not be a strategic priority, especially for standalone voluntary organisations who largely rely on charitable funding to fund patient care [23]. Palliative care research can be time consuming and staff may see it is an ‘add on’ to their role and not part of the routine care they provide to patients [24]. Staff may feel that they lack the necessary knowledge, skills and expertise to be involved in palliative care research [4, 25] and may have limited opportunity to participate or learn more, especially when balancing clinical pressures that have increased during the COVID 19 pandemic [26]. An organisational research culture improves outcomes for all patients, and not just those involved in the research [27]. The aim of this study therefore is to further understand professional and organisational facilitators and barriers to conducting all types of palliative care research.

## Methods

### Research question

What are the barriers and facilitators to conducting palliative and end-of-life care research across North West Coast England*?*

### Design

A mixed method study following a convergent design [28]***,*** incorporating a cross-sectional online survey and working groups using a nominal group technique [29]. The survey is reported according to the CHERRIES guidelines for e-surveys [30].

### Setting

Both the survey and working groups were conducted across the UK NIHR North West Coast region of England (incorporating South Cumbria, Lancashire, Cheshire, and Merseyside). Currently, palliative care research activity within this area is low. In the UK, palliative care is provided by generalists, the patient’s usual care team, in the hospital, community or care home setting. Specialist inpatient, hospital, home and home nursing palliative services are provided by professionals specifically trained in palliative care, and they largely rely on charitable funding [31, 32].

### Population

All those who had interest in the provision of, or research into, generalist or specialist palliative care across the region including across acute and community NHS Trusts, GP practices, voluntary hospices, other community and private providers of care, clinical research networks, and academic settings including Universities were invited to participate. The survey was accessed via an online link that included a screening question incorporating the inclusion criteria (see Table 1).

**Table 1:** Study inclusion criteria

Inclusion criteria
Provide health and/or social care for patients and carers (adults and/or children) with palliative/end of life care needs *and/or* Involved or would wish to be involved in palliative/end of life care research
Aged 18 + , no maximum age
Working within the North West Coast geography (South Cumbria, Lancashire, Cheshire, and Merseyside)

### Sample

*Survey:* The survey used a convenience sampling approach and was designed to collect largely descriptive data and yield rich information across a range of respondents. Without a viable sampling frame of potential participants, no anticipated sample size could be reliably estimated. *Working groups*: Those who indicated an interest in taking part via their survey response, or who responded to additional calls for participation, were invited to participate, and then purposively selected to maximise variability across professional background, expertise, and geography.

### Recruitment

*Survey:* Potential participants were recruited via several routes that included dissemination via collaborators in local NHS Trusts and Hospices and the North West Coast Clinical Research network to ensure primary care organisations were reached. Information about the survey was openly and widely disseminated through a project website, personal networks, and social media (Twitter, Facebook, and LinkedIn). No incentives for survey completion were offered. Dissemination included a link to the online survey, with screening questions at the start of the survey confirming eligibility, with clicking through to progress to the survey indicating consent. Potential participants were reassured that taking part was voluntary and that survey results would be aggregated and anonymised. It was explained that their data would be inputted into a secure online survey platform, and these data would be then stored in a secure institutional filestore at Lancaster University. (see additional file 1).

### Working groups

Individuals who expressed an interest in taking part in further research after completing the survey were sent working group invitation packs. Additionally, collaborators in local NHS Trusts, Hospices and the North West Coast Clinical Research network circulated packs to eligible participants. Social media (Twitter, Facebook, and Instagram) was also used to advertise the working groups. Participants could take part in the working groups even if they had not completed the survey. Participants contacted the research team if they were interested in taking part and electronic consent was obtained prior to the working group.

### Data collection

*Survey:* The open online survey was built using Qualtrics^XM^ [33], and the full survey is included in additional file 1. Both closed and free-text questions were used, together with skip options dependent on given answers; 19 possible questions (some with multiple components) were asked across 5 blocks. Participants could navigate through the survey using forward and back buttons. The survey identified current and desired levels of palliative care research involvement, current research barriers, suggestions for sustainable solutions and research training needs. The survey was developed from the IPOS survey (a survey of the research barriers and training needs within the International Psycho-Oncology Society) [34] and literature on barriers and facilitators to palliative care research [3]. Survey development followed an iterative approach, with members and colleagues of the project steering committee reviewing survey questions to ensure the survey was appropriate. Participants could only complete the survey once. There was not a completeness check for respondents. The survey was open from 02/03/2022 to 08/06/2022.

### Working groups

Four online (via Microsoft Teams) working groups took place. The groups lasted two hours and were facilitated by LD and another member of the research team (from CW, AG, BS, RB). Nominal group technique was used as it is a method that elicits the views and opinions of a group of experts through the ranking of priorities related to a particular topic of interest. It combines both qualitative and quantitative data collection and involves a number of stages that include; introductions, silent generation of ideas, listing of ideas, discussion of ideas, ranking of top ten ideas, voting on top ten ideas, discussion of voting and conclusions [29]. Mentimeter [35] was used to facilitate the voting process and the working groups were recorded.

### Data analysis

*Survey:* Data were downloaded from Qualtrics™ as.csv and.sav files for Excel and SPSS, hosted on Lancaster University secure OneDrive, and checked for potential duplicate entries (using IP, email address or organisation name to ensure only one entry per respondent), and to remove incomplete entries. Entries were judged as complete when participants had provided sufficient descriptive personal information alongside survey responses, even if answers to all available questions had not been given. Pseudonymised data were used for analysis. Descriptive analysis included the use of frequency counts, percentages, and rankings, with some collapsing of categories.

For the analysis of free-text comments, data were extracted into Microsoft Excel. Comments tended to be brief, expanding on answers to closed questions [36, 37]. After initial familiarisation, a coding framework was inductively developed by LD and CW and applied to the free text data using a conventional content analysis technique [38]. Coding and theme development were driven by the content of the free-text comments.

### Working groups, using nominal group technique

Each working group was initially analysed separately by LD using the group’s Mentimeter rankings as an initial a priori framework [39]. The working group recordings and transcripts were read and listened to, and the key issues were summarised within the a priori frameworks. The findings were then compared across the working groups by LD, SM, BS, and AP with input from the study’s Patient and Public Involvement group and finally the study steering committee, to identify key themes.

Four overarching groupings were inductively generated after completion of the working groups. Survey free text and working group findings were compared as part of the four theme development. Mentimeter rankings were allocated to the four groups along with the survey statements where there was strongest agreement about the barriers to research across all survey respondents (see Table 5.).

### Ethics

Approval was granted by the East of England—Cambridge South Research Ethics Committee (Ref: 22/EE/0049) on the 24/02/2022. Organisational approval was obtained via the Health Research Authority and each participating site.

## Results

### Survey response

The online survey received 495 visitors, of whom 8 declared they did not meet the inclusion criteria, 36 provided no data, and 158 did not proceed beyond the screening questions. Valid responses were received from 293 participants (59% of visitors), with 171 of the 293 (58%) recording 100% survey progress, and a mean progress of 82% (range 100% to 25%).

### Characteristics of survey respondents

Full descriptive data from these respondents are found in Table 2. The highest proportion of respondents worked in hospice settings, were nurses, and had worked in palliative care for over 10 years. Unexpectedly, there was a high number of paramedics who completed the survey (*n* = 17).

**Table 2:** Characteristics of survey respondents

Characteristic	Number *N* = 293	Percentage
Work setting	n	%
Hospice	78	27%
Hospital	69	24%
Primary Care	58	20%
University	18	6%
NHS R&D	17	6%
Clinical Research Network	5	2%
Nursing/Care home	2	1%
Other^a^	44	15%
Missing	2	1%
Professional background	n	%
Nurse	133	45%
Doctor	48	16%
Researcher	24	8%
Physiotherapist	4	1%
Manager/Admin	24	8%
Social Worker	5	2%
Occupational Therapist	7	2%
Other^b^	48	16%
Length of time working in palliative care	n	%
< 2 years	44	16%
2 to 5 years	51	18%
6 to 10 years	61	22%
10 + years	128	45%
Missing	9	3%
Work in specialist or generalist palliative care	n	%
Specialist	113	39%
Generalist	68	24%
Research only	22	8%
Other^c^	41	14%
Not applicable	43	15%
Missing	6	2%
Work with adults or children	n	%
Primarily adults	270	95%
Primarily children	14	5%
Missing	9	3%

Research experience or roles of those completing the survey are presented in Table 3. Nearly 75% of respondents were not active in research, but nearly 73% wanted to increase the time they spent on research

^a^community setting 27, emergency/ambulance/pre-hospital setting 13, multiple settings ≤ 5,

^b^paramedic 17, health care assistant 7, pastoral support worker 6, research practitioner 6, manager ≤ 5, pharmacist: ≤ 5, speech and language therapist: ≤ 5, nurse specialist: ≤ 5 student: ≤ 5 PPI: ≤ 5

^c^miscellaneous 18, primary/community care 9, emergency care 7, acute care 5, education ≤ 5

**Table 3:** Research experience and role characteristics of survey participants

Characteristic	Number *N* = 293	Percentage
Palliative care research experience	n	%
Non-active	199	75%
Involved	47	18%
Managing	11	4%
Supervising	9	3%
Missing	27	9%
Proportion of time spent on palliative care research	n	%
None	184	68%
Less than 10%	59	22%
10–25%	13	5%
26% +	14	5%
Missing	23	8%
Would you like to increase the time you spend on research?	n	%
Yes	197	73%
No	74	27%
Missing	22	8%
Number of current research projects	n	%
None	230	86%
1 to 3	31	12%
4 +	6	2.3%
Missing	26	9%

### Characteristics of working group participants

Twenty palliative care providers/research staff participated in the working groups (see Table 4 for details).

**Table 4:** Staff working groups participant demographic details

	Characteristics	Number (*N* = 20)	Percentage
Working group attended	Working group 1 Working group 2 Working group 3 Working group 4	5 7 5 3	25% 35% 25% 15%
Gender	Female Male	15 5	75% 25%
Age in years	18–30 31–40 41–50 51–60 60 +	1 7 5 5 2	5% 35% 25% 25% 10%
Ethnicity	White Black/African/Caribbean/ Black British Other	18 1 1 0	90% 5% 5%
Length of time worked in palliative care	Just getting started (< 2 years) Early career (2–5 years) Mid-career (6–10 years) Late career (10 + years)	0 7 4 9	0 35% 20% 45%
Primary professional role	Nurse Doctor Manager/admin Other	9 5 3 3	45% 25% 15% 15%
Primary work environment	University Hospital Primary Care Hospice Ambulance Trust	2 2 2 13 1	10% 10% 10% 65% 5%
Role	Specialist palliative care Research role only Other primary role Missing data	12 2 4 2	60% 10% 20% 10%
Primary population cared for	Adults Children	16 4	80% 20%

### Barriers and facilitators to participating in palliative care research (quantitative data)

Survey respondents were asked to indicate the strength of agreement with statements about facilitators or barriers to engagement and involvement with palliative care research. Working group participants inductively generated statements about barriers which were then ranked. In Table 5. below we present the survey statements where there was strongest agreement across all survey respondents, together with the ranking of inductively generated statements from each of the working groups. Full survey data are found in additional file 2.

**Table 5:**
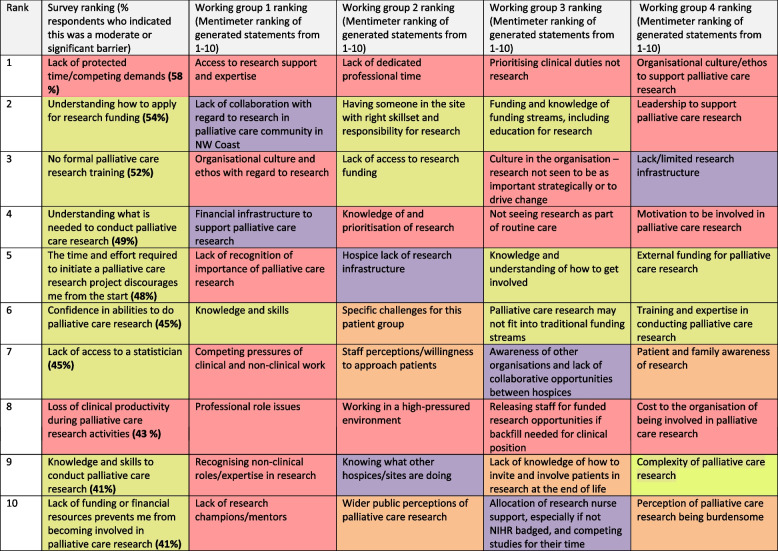
The top ten barriers to participating in research identified from the survey and each of four working groups

The top research barriers were conceptualised across four main areas: organisational culture and capacity (including prioritisation and time given to research); research knowledge (including research skills, how to obtain funding); research infrastructure and collaborations (including collaborative opportunities and governance arrangements), and patient and public perceptions of palliative care research (including vulnerabilities and burdens). Data on facilitators and training needs were collected in the online survey and are presented in Tables 6 and 7.

**Table 6:** Top 10 facilitators to participating in palliative care research (combined agree or strongly agree from survey data)

Top 10 facilitators to participating in palliative care research	% who indicated this was a combined agree or strongly agree
Palliative care research information network	62% *n* = 181
Palliative care research seminars for those in practice	62% *n* = 181
Collaboration with other centres	62% *n* = 181
Availability of resources such as a guide/manual	61% *n* = 179
Supportive management	61% *n* = 178
Staff cover	61% *n* = 178
Access to funding to support research	61% *n* = 178
Palliative care research mentors’ programme	60% *n* = 177
Attending research conferences	60% *n* = 177
Access to allocated research staff	60% *n* = 177

**Table 7:** Top 10 training needs from the survey

Training Needs	Least Interested	Moderately Interested	Most Interested	Missing
Using research data to inform programmes and services	7%	57%	26%	41%
Designing palliative care research studies	11%	27%	23%	39%
Identifying research mentors	11%	26%	22%	41%
How to design rigorous and evidence-based research while being pragmatic and taking into account the complex environment in which palliative care research is often carried out	12%	28%	21%	39%
Qualitative research designs	12%	30%	20%	40%
Qualitative data collection (e.g., focus groups, interviews)	8%	31%	20%	41%
Writing a successful grant application	18%	20%	20%	42%
Developing a programme of research	11%	29%	19%	41%
Project management	11%	30%	18%	41%
Finding grant funding	19%	21%	18%	42%

### Barriers to participating in palliative care research (qualitative data)

Additional data on the four areas of organisational culture and capacity, research knowledge, research infrastructure and collaborations, and patient and public perceptions of research were generated in both the free text comments from the survey and working group analysis. A narrative exploring each of these is presented in turn, illustrated with verbatim data extracts from the working groups and survey.

#### Organisational culture and capacity

This was the top barrier identified in the survey and most working groups. The focus was about whether research is prioritised within the organisation, including if people are enabled to conduct research in terms of protected time. Across the working groups and survey, participants explained how staff have no time to be involved in research because of clinical pressures and commitments. Staffing shortages, patient complexity, and the impact of COVID 19 have made the situation even more challenging for clinicians:

*‘It's really difficult because everyone is so stretched that everybody's so busy sort of, you know, the AHP's [allied health professionals], the doctors, the nurses, everyone's very busy, sort of fighting fires that nobody's got time to move away from that at the moment’ (Hospice Doctor, working group 2)*

*‘The main barrier from my experience is not having protected time to spend in research activities. My case load is vast and give me no time to participate in research. This is disheartening to me as we need to constantly develop and not stagnate. Also, with palliative care we get one opportunity to make that difference so we need to be equipped with the best we can do.’ (Survey study ID 163, Hospital Doctor)*

Organisational culture and external requirements also mitigate against engagement in palliative care research, where priority is given to meeting key performance indicators, which rarely include research engagement:

*‘The clinical demands and their key performance indicators required by our service specifications and our trust, demand that you spend the majority of your time 90% if not more, undertaking clinical aspects of the role and that there isn't necessarily buy in [to research] I don't feel from the senior management within the organisation to support us’ (Palliative care nurse specialist, working group 1)*

Research not being part of an organisations culture and ethos and therefore not seen as a strategic priority was an important barrier.

*‘Even if someone said here's some funding, what do you want? We reel off a million answers, but research would probably be at the bottom just because there's other things that we need or want that we feel is probably more important than research. Whether that's right or wrong, I think it's just not. Not a priority. It's no one’s first thought.’ (Hospice nurse, Working group 2)*

Participants highlighted the need for a ‘research champion’ within an organisation who would be responsible for leading, prioritising and raising the profile of research therefore making research less daunting for staff.

*‘I think you're somebody who's motivated to drive a research agenda forward, I think makes a big difference in the organisation that you're in, whether that's hospital based or community Hospice and based because I think if you haven't got anybody who's keen and enthusiastic, you're not going to go anywhere. So you've got to have someone who's willing to take that on.’ (Hospital Doctor, Working group 4)*

#### Research knowledge

Health and social care staff can have a limited understanding of research processes, and therefore may not have the necessary skills to conduct research. Whilst some basic knowledge was covered at pre- and post-registration undergraduate or postgraduate level, continuing to develop skills and knowledge could be challenging:

*‘We're encouraging our staff to undertake further education or sort of masters level qualifications, and at that level it does require for the qualification a piece of research and a number of research questions to be undertaken, but it's how do we move beyond that?’ (Hospice manager/admin Working group 1)*

*You do the research project within the course to get through the course and then you know you like, breathe sigh of relief and then you don't go near research again.’ (Palliative care nurse specialist, Working group 1)*

Research can feel distant and overwhelming, academic and jargon filled, without relevant pathways to support professional development:

*‘I think from a perspective of peoples understanding and knowledge of research and where to get support and there's a lot of people shy away from it because they don't know where to start. They don't know where to go to. They don't know how to find the literature and they just feel like they're in a minefield of information they don't know which avenue to take.’ (Hospice nurse, Working group 4)*

The need for mentorship, support, and guidance from more experienced research staff and how to access this support was clearly identified. Engaging junior staff was seen as important and training sessions/e-learning needed to be accessible, including tailored resources for palliative care, and level of involvement in research.

*‘If people haven't done a lot of research and they want to be involved and it's sort of supporting that group of people if they haven't got links to people already or groups within their organization or network that they can link into, and they're really interested in it, it's getting those people involved and how to direct them?’ (Hospice nurse, Working group 4)*

*‘Need the support of an experienced researcher and also someone to help plan and develop the research, mentor and guide throughout research project and assist with analysis of results-/stats and writing up the project.’ (Survey study ID 39 specialist palliative care clinical manager)*

Participants explained how there tended to be a lack of research expertise (e.g. knowledge of research processes) within hospices and how it was important to have someone with the right skill set in the setting/small organisation.

*‘Having somebody with the right skill set to take something through ethics committee and everything I suppose, and you need to have that one person in every Hospice or in every setting who can do all that. It's a skill all of its own.’ (Manager/admin, Working group 2)*

#### Research infrastructure and collaborations

Palliative care research was felt to have a weak infrastructure, with few studies in the National Institute for Health Research (NIHR) portfolio, limiting opportunities to be involved in research and access to research nurse support. Hospices had few financial resources to support research activity, and seemed reluctant to divert funds from direct patient care:

*‘So, there's huge financial implications in terms of them [charitably funded hospices] providing sort of and delivering research … it was a massive competing pressure on money because you don't want to be impacting on the organisations finances and within the charity sector to the detriment of immediate patient care.’ (Hospice Doctor, working group 1*)

*‘Releasing people to take part in research is just impossible for a Hospice with our current funding arrangements. Research feels like a "nice to have" aspect of Hospice work. Even though I know it would be valuable to our sector long-term to be research active, the climate we find ourselves in means research is way down the list of priorities for a charity receiving 30% (and diminishing) funding* [from the NHS] *to run a 24/7 service.’ (Survey Study ID 85, Hospice CEO)*

The lack of or limited research infrastructure outside the hospital setting, particularly within standalone hospices, was raised as a barrier. The necessary structures to support research activity, such as governance arrangements, training, and adequate staffing levels, could often be lacking.

*‘I think when you're working with within small groups you could be quite isolated with only having one research nurse who then is on their own, and I think the link I think that's probably an issue in terms of I guess the funding for that person. It can be an issue but also attracting somebody to a post which is going to feel quite isolating.’ (Hospital Doctor, Working group 4)*

*‘But the thought of actually undertaking some research ourselves. We're a million miles away from that in our hospice you know. We are trying to be involved in other bigger trials, but where to actually put through an ethical approval ourselves. We're nowhere near that here.’ (Hospice Doctor, Working group 2)*

The importance of engaging nursing and allied health professionals in research and giving them the opportunity to be involved was raised. The four pillars of professional practice of the clinical nurse specialist and advanced practitioner roles includes research alongside clinical, education and leadership components [40]. However, research is not always recognised or developed. It was noted that organisations support training in Independent and Supplementary Prescribing, diagnostics, and advanced communication skills, so it was questioned why not research. Some short-term research positions may not provide opportunities for all staff, as posts may be linked to certain roles (e.g. medical, nursing) or require professional registrations, thus limiting opportunities for staff without these qualifications (e.g. healthcare assistants). The importance of recognising the role and expertise of non-clinical staff in research and its potential impact on care and services needs to be promoted.

Currently, there was not a strong sense that people or organisations were working collaboratively locally or regionally to facilitate research:

*‘We don't work collaboratively, and we have a really big list of research projects that we'd like to do. We'd like to get started on. We don't have the capacity to do it, but actually other hospices or other professionals in palliative care might be working on it. But we just don't know because we don't talk to each other. Perhaps we just need to talk more?’ (Manager/admin Working group 2)*

*‘I think we're all busy, aren't we? So, the opportunity to meet, collaborate, share ideas doesn't to me seem like it's there. I could be wrong, but I think lack of existing collaboration, just perhaps due to how busy we all are individually, and rather than what I didn't mean, was competitiveness between hospices, yeah.’ (Hospice nurse, Working group 3)*

*‘From a researcher perspective, the barriers I face are around making the necessary connections with relevant practitioners interested and available to work on research projects. This is partly to do with few opportunities to meet people in informal environments where research priorities or interests can be discussed….(Survey study ID 43 researcher)*

The need for some form of alliance or collaborative infrastructure was highlighted to pool research ideas, share information, collaborate on policies and governance issues. This was felt to need buy in from multiple organisations, potentially with a funded post to lead on research across voluntary hospices:

*‘it's almost like we need some sort of alliance, isn't it? And that may well be where all this is headed and in terms of, you know, somewhere in the region somebody's putting a bid in for this research and who wants to jump on board to recruit in their area to get some opportunity for the expertise.’ (Palliative care nurse specialist, Working group 1)*

*‘And so maybe having some kind of umbrella group or network that… then everything kind of filters through it and information comes back out the other way so that that information is shared and you kind of know where to go. Maybe if you've got an idea to check that no one else is already doing it and to be in touch with the right people at the right time, I don't know if something around the kind of coordination of the whole thing.’ (Hospice manager/admin, Working group 2)*

There were concerns raised that the palliative care research community involved a select group of individuals and could be elitist. It could be difficult for those sitting outside the elite to know how to be involved and included in any research activity:

*‘I did reflect on initially when I got interested in research it was sort of seen as this area of expertise in which a select group were involved, and it was sort of how do we get into that Network.’ (Hospice nurse, Working group 4)*

#### Patient and public perceptions of palliative care research

Concerns were also raised that patient and public perceptions of palliative care research may be an issue either because there were assumptions that research was not happening, or only in large/cancer settings, that people did not want to take part, or that the end of life is an inappropriate time to request participation.

*‘Sometimes staff feel oversensitive. Almost oversensitive to not wanting to upset patients and relatives to recruit them in, or to ask the relevant questions that we need them to ask.’ (Hospice educator, Working group 2)*

However, counter arguments were also recognised:

*‘Anecdotally, we've had people tell us when they've taken part in studies that we've done, that they've enjoyed taking part that it's been beneficial for them, not because the research will impact them, but because of the process of...I guess the therapeutic aspect that's a side line to them taking part that they've enjoyed taking part and sharing. Their views and being able to put something back and to help other people.’ (Researcher, Working group 3)*

## Discussion

### Summary

The aim of this research is to understand professional and organisational facilitators and barriers to conducting palliative care research. Palliative care research was recognised as important and valuable, with three-quarters of those involved in this study wanting to increase their involvement in research, despite most not being currently research active. Several key barriers to palliative care research were identified including lack of organisational research culture and capacity (including prioritisation and available time); research knowledge (including skills/expertise and funding opportunities); research infrastructure and collaboration (including lack of collaborative opportunities across multiple organisations and governance challenges); and patient and public perceptions of research (including vulnerabilities and burdens). Key facilitators included dedicated research staff, and active research groups, collaborations, and networking opportunities.

### What this research adds

A key finding is the apparent lack of progress in facilitating palliative care research over time, and the challenge for the sector is why change has been so slow. Previous palliative care research identifies a suite of remarkably similar barriers [23, 41–44], albeit not necessarily unique to this specialty [45, 46]. There needs to be a concerted and sustained focus on collaboration and sharing best practice, developing a research culture and facilitating research within and between palliative care providers, enhancing staff capacity and expertise, and providing guidance on research processes and procedures [23, 41, 43, 44]. Our research further highlights the importance of organisational barriers, pointing to the need to prioritise organisational solutions.

Organisations have a critical role in building research culture and capacity [46–48]. It is imperative that organisations recognise and value research and incorporate research into the core business of the organisation. This means that research should be visible throughout, from mission statements to policies, business plans, and job descriptions. They should protect research time and resources, recognise talent, and reward positive research related behaviours [48]. This may be a particular challenge for those palliative care organisations that are charitably funded due to the uncertainty and volatility of their funding [49, 50], and business models that may not account for research activity [51]. The focus is also set nationally, with the recently launched Hospice UK 2024–29 strategy having no overt mention of research [52].

A key finding is that for many the organisational lack of support for research translates into research not being seen as a core part of people’s jobs. Again, this is not unique to palliative care, with capacity to be engaged in research limited in time or job plans [53]. As an example an audit of clinical nurse specialist job descriptions found that 80% had an expectation of research engagement [40], however, in detailed studies of how such roles are enacted, research is typically absent [54, 55]. Where research is mentioned, it was in the context of it being the least important aspect of the role, or that others (such as medical consultants) should be leading research [56]. However, whilst there is little contemporary data, previously the median time palliative care consultant doctors spent on research was zero hours [57]. A recent survey of UK palliative medicine consultants found that while 78% (*n* = 140/180) were interested in conducting research, 83% had no allocated time within their job plan [58]. Given the serious and significant workforce pressures and challenges currently facing many healthcare workers it is unlikely this position will change without both investment in, and prioritisation of, research time and roles. It may be that research time or engagement needs to explicitly form part of key performance indicators or other metrics to enable such prioritisation to occur.

Research should be important to palliative care provider organisations. It is known that a strong research culture and organisational research performance lowers mortality rates, increases patient and staff satisfaction, reduces staff turnover, and improves organisational efficiency [59]. Our research encompassed a variety of different organisations and settings, demonstrating that these barriers were remarkably similar wherever a person worked. Solutions may differ though depending on the size, funding, and specialism of the organisation. An independent voluntary funded hospice may have different solutions to a palliative care team working as part of a larger general hospital or community care provider.

The opportunity to collaborate between individuals and across organisations may be important, as in other specialities such as General Practice [60]. Evidence indicates that the creation of research cooperatives, collaborations and partnerships can be fruitful. There are palliative care examples from the UK [61], US [62, 63], Australia [64–66], and Africa [67]. Some of these are large collaboratives, across multiple sites, facilitating multiple studies [68]. It is possible that such collaboratives mitigate the effect of the employing organisation for members, facilitating research in a way that sits above, and possibly either bypasses, negates, or gives the skills to overcome institutional and local organisational barriers. Joint approaches between universities and public and charitable providers of palliative care may help overcome structural issues such as indemnity, sponsorship and gaining research ethics committee approvals. However, funding to sustain some of these collaborations can be fragile or time limited. For example, in the UK, very welcome but time-limited funding to build palliative care research partnerships has been awarded, but it is too early to see the impact of this on the research landscape [69]. The benefits of such collaborations may also be on the wider research culture of the organisations that participate in such research. The initial impact of participating in a trial may be staff stress and workload, but this has found to be replaced by enthusiasm for the changes and benefits achieved [70].

Those who completed our survey had wide variability in levels of research experience and involvement. It is important to recognise when considering developing an organisational research culture that not all members of staff need the same level of skill and expertise, and not all organisations will be at the same level of engagement. Previous recommendations for hospices suggested a typology of engagement, through which hospices could progress if they wished, from research aware, to research engaged, to research leading [23, 43]. Equally, individuals can have different levels of preparation, with recognition that generating and leading new research likely needs the higher levels of research preparation such as research focused PhDs, and that organisations that aspire to these levels need to invest in educating staff to these levels and supporting their continued research development.

### Strengths and limitations of the research

A strength of this research was the breadth of response from across different sectors and professional backgrounds. There was a particularly strong response from nurses, and a reasonable proportion of those providing general palliative care. However, it was harder to recruit respondents who do not provide specialist palliative care (perhaps because they do not identify themselves as palliative care providers despite the high numbers of those with palliative care needs that they provide care for). Care home respondents were particularly poorly represented. We aimed to invite patients, family members and the public to a working group. Whilst we involved Patient and Public Involvement (PPI) study team members in planning this work and attempted to recruit the public to our working groups, challenges both in institutional permissions and recruitment meant that this planned aspect of the study did not go ahead. This work also represents the views of people from across a particular UK geography. Whilst this includes a large, diverse, population it may be that this does not represent wider views, although this is unlikely given the congruence with past and related research. This study also includes participants who were involved or would wish to be involved in palliative care research so the views of those who are not interested are not reflected in the findings.

## Conclusions

Engagement in palliative care research appears stagnant, with this study revealing a range of barriers that appear unchanged from a decade or more ago. The challenge for palliative care is not to identify further the barriers and facilitators to research, but to invest time and funding to address the known barriers and enable the facilitators of research. It is likely that such investments will reap dividends in terms of staff satisfaction, organisational performance, and importantly the quality of care provided to patients and families.

### Supplementary Information

Supplementary Material 1.

Supplementary Material 2.

## Data Availability

Data are stored in Lancaster University’s PURE repository, consent to share data was not given by participants.
