# Supplementary material for: Understanding barriers and facilitators to palliative and end-of-life care research: a mixed method study of generalist and specialist health, social care, and research professionals
Source: BMC Palliat Care. 2024 Jun 25;23:159. doi: 10.1186/s12904-024-01488-2 (PMC11202245; doi:10.1186/s12904-024-01488-2)
Supplement: Supplementary file 2 — Supplementary Material 2. [file 12904_2024_1488_MOESM2_ESM.docx]

**Additional file 2: Full survey findings** (In the tables below, the green highlights are the top 10 rankings, including tied rankings.)

**List of barriers (full sample)**
